# Supplementary figures and images for: Longitudinal assessment of fluorescence stability shows fluorescence intensity decreases over time: implications for fluorescence microscopy studies
Source: PLoS One. 2026 Mar 5;21(3):e0343635. doi: 10.1371/journal.pone.0343635 (PMC12962454; doi:10.1371/journal.pone.0343635)

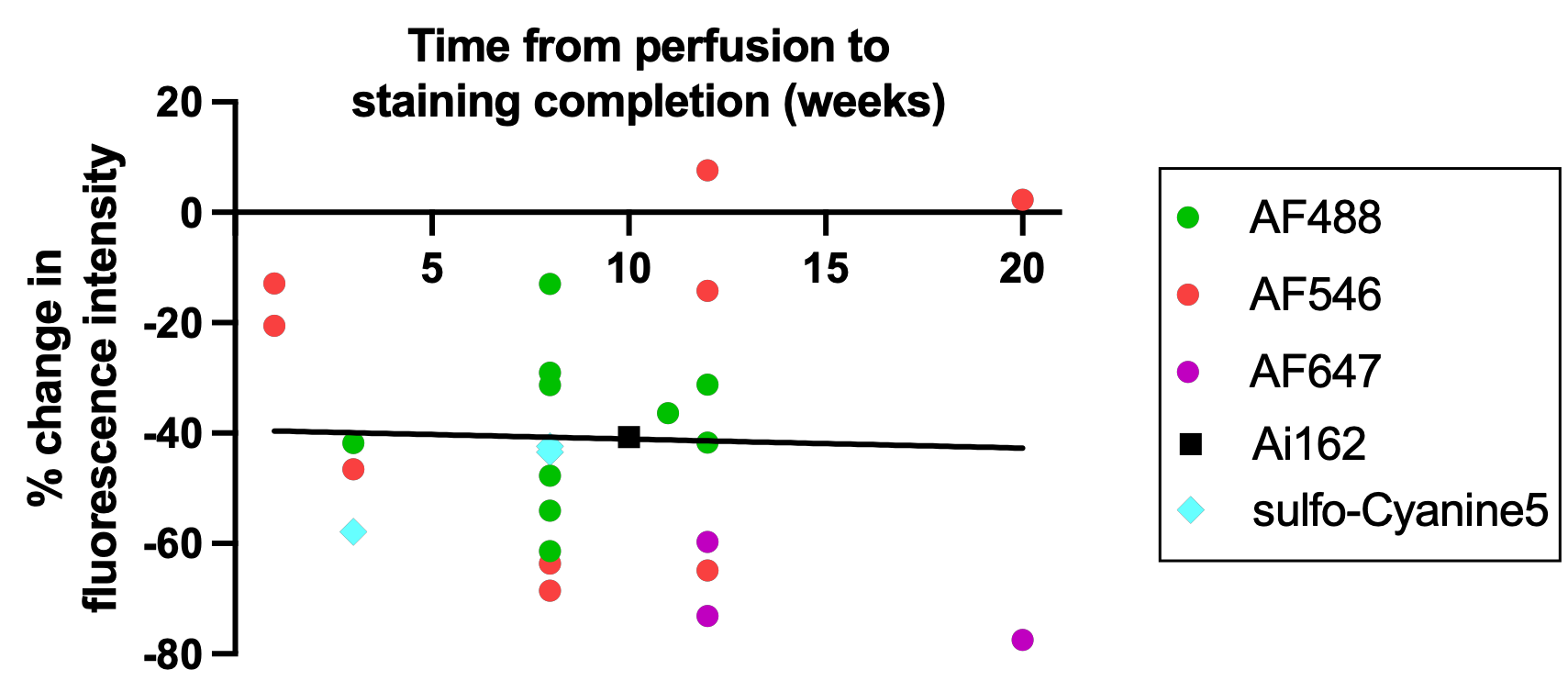

Supplement: S1 Fig — % change in fluorescence intensity between the first and last imaging time point is shown relative to the time between perfusion and staining completion for that sample. All data sets with either four weeks (confocal imaging) or six weeks (widefield imaging) between the first and last imaging time point were included. Hence, all data in the manuscript except for Ctrl Exp. 1 (daily imaging for one week) and Ctrl Exp. 3 (imaging every 2 min for 7 time points) are included. Linear regression analysis of all plotted data showed no relationship between time from perfusion to staining completion and the percentage changes in fluorescence intensity over the 4–6-week imaging time period (y = −0.16x −39.4, R2 = 0.001, p = 0.87, F(1,24)=0.028, n = 26 [10 AF488, 9 AF546, 3 AF647, 1 Ai162, 3 sulfo-Cyanine5]). (TIFF) [file pone.0343635.s001.tiff]
